# Genetics of Recurrent Vertigo and Vestibular Disorders

**DOI:** 10.2174/138920211797248600

**Published:** 2011-09

**Authors:** Irene Gazquez, Jose A Lopez-Escamez

**Affiliations:** 1 Otology & Neurotology Group, CTS495, Centro de Genómica e Investigación Oncológica –GENyO Pfizer-Universidad de Granada- Junta de Andalucia, Granada; 2 Department of Otolaryngology, Hospital de Poniente, El Ejido, Almería, Spain

**Keywords:** Vestibulopathies, recurrent vertigo, Meniere’s disease, genome-association studies.

## Abstract

We present recent advances in the genetics of recurrent vertigo, including familial episodic ataxias, migraneous vertigo, bilateral vestibular hypofunction and Meniere’s disease.

Although several vestibular disorders are more common within families, the genetics of vestibulopathies is largely not known. Genetic loci and clinical features of familial episodic ataxias have been defined in linkage disequilibrium studies with mutations in neuronal genes KCNA1 and CACNA1A. Migrainous vertigo is a clinical disorder with a high comorbidity within families much more common in females with overlapping features with episodic ataxia and migraine. Bilateral vestibular hypofunction is a heterogeneous clinical group defined by episodes of vertigo leading to progressive loss of vestibular function which also can include migraine. Meniere’s disease is a clinical syndrome characterized by spontaneous episodes of recurrent vertigo, sensorineural hearing loss, tinnitus and aural fullness and familial Meniere’s disease in around 10-20% of cases. An international collaborative effort to define the clinical phenotype and recruiting patients with migrainous vertigo and Meniere’s disease is ongoing for genome-wide association studies.

## INTRODUCTION

Genomic medicine in complex middle or inner ear diseases such as chronic otitis media, otosclerosis or age-related hearing loss is an emerging topic [1]. Patients with imbalance or recurrent vertigo are a heterogeneous group of complex disorders affecting the peripheral and central vestibular system and they represent a diagnostic challenge for the clinicians since their genetic basis is largely not known. Familiar episodic ataxias are rare cause of recurrent vertigo, but molecular genetics has identified several mutations on KCNA1 and CACNA1A genes that suggest a key role for voltage-gated channels and solute carriers in the plasma membrane of neurons in recurrent vertigo. Moreover, recent advances in familiar vestibular disorders and particularly in familial Menière’s disease have facilitated the pathways for clinical characterization and recruitment of patients for large collaborative studies.

Recurrent vertigo with vestibular hypofunction is a common symptom in clinical practice and there is a hereditary component in neurotologic disorders that is not well defined. Migraine has a strong genetic background and it is frequently observed in these patients and although the genomics for migraine has started to become known [2, 3], the shared allelic variants between vertigo and migraine remain to be discovered. This review will include syndromes with recurrent or episodic vertigo: familial episodic ataxia, migrainous vertigo, bilateral vestibular hypofunction and Ménière’s disease.

## FAMILIAL EPISODIC ATAXIAS

Familial episodic ataxias are monogenic recurrent vertigo syndromes. Most of them are autosomal dominant disorders of early onset characterized by recurrent attacks of incoordination, dysarthria and truncal ataxia. There are several subtypes defined by associated interictal findings and genetic characterizations (Table **1**). The key clinical feature that raises the diagnosis of episodic ataxia is discrete attacks of incoordination with a clear onset and resolution of symptoms, which also distinguishes episodic ataxia with progressive features from progressive ataxia with intermittent exacerbation. The presence of interictal findings in most patients provides a helpful distinction with vestibular migraine.

### Episodic Ataxia Type 1 (EA1)

EA1 is a potassium channelopathy characterized by constant myokymia and episodes of spastic contractions of muscles of the head, arms and legs. The attacks consist of episodes of vertigo lasting minutes associated with diplopia, headache, stiffness of the body and dysarthric speech.

EA1 is caused by mutations in KCNA1 gene located on chromosome 12p13 (MIM 160120), which encodes Kv1.1, a voltage-gated potassium channel [4]. Functional Kv1.1 channels are tetrameric structures composed of four identical monomers, but K+ channel diversity is greatly enhanced by the ability to form heteromeric channels composed of Kv1.1 and Kv1.2/Kv1.4/KvB1.1. In several brain areas, Kv1.1 co-assembles with Kv1.4 which confers N-type inactivating properties to heteromeric channels. It is likely that the rate of inactivation will be determined by the number of Kv1.4 subunits which is increased in EA1 [4], resulting in an increase in neuronal excitability [5, 6]. Individuals with EA1 are heterozygous for a KCNA1 disease-causing mutation, and they have a wild-type and a mutated allele, which may be equally expressed; so, channels composed of wild-type and mutated subunits may be formed, but this heterozygous allele is enough to alter the function of heteromeric channels containing Kv1.1 subunits [7, 8].

The KCNA1 gene has a transcript of 7983 nucleotides with a coding region of 1488 [7]. To date, more than 20 KCNA1 mutations have been described and most are missense mutations distributed throughout the gene [9]. Interestingly, four different mutations of the highly conserved threonine 226 in the second transmembrane segment (T226M/A/R/K) that lead to diverse phenotype have been identified [10].

### Episodic Ataxia Type 2 (EA2)

EA2 is a Ca^2+^ channelopathy characterized by recurrent vertigo lasting from hours to days, with imbalance, vomiting, ataxia with interictal nystagmus (MIM 108500). It is distinguished from EA1 by the absence of myokymia (fine rippling of muscles). Episodes are triggered by exercise, an intercurrent infection, stress, alcohol and caffeine and they are often relieved by treatment with the carbonic anhydrase inhibitor, acetazolamide [11]. Migraine occurs in about 50% of patients with EA2.

EA2 is caused by mutations in the CACNA1A gene, mapped to chromosome 19p13 [12], which encodes the α-subunit of the P/Q-type voltage-gated calcium channel, Ca_v_2.1 [13, 14]. This gene is widely expressed throughout the CNS, showing highest levels at Purkinje and granule cells of the cerebellum. 

Dominant mutations in CACNA1A underlie at least three allelic diseases: EA2, familial hemiplegic migraine type 1 and spinocerebellar ataxia type 6. A large number of different single nucleotide mutations have been shown to cause EA2 which results in premature stop codons and a non functional protein with loss of Ca_v_2.1 channel function [15, 16], including recent mutations in the promoter and a new final exon 48 [16]. Moreover, these mutant subunits may disrupt the membrane trafficking of wild-type subunits [17]. CACNA1A alternative splicing in the cerebellum of selected exons harboring nonsense mutations (such as exon 37A) can also cause EA2 [18]. Direct sequencing of CACNA1A in some patients with EA2 does not identify any point mutation, but the use of methods such as MLPA (multiplex ligation-dependent probe amplification) and QMPSF (quantitative multiplex PCR of short fluorescent fragments) has demonstrated large-scale CACNA1A gene rearrangements (deletions and duplications) in patients with EA2 [19,20].

Familial hemiplegic migraine type 1 is a form of migraine with aura and reversible hemiparesis also caused by missense nucleotide mutations in the CACNA1A gene that alter channel gating and enhance the channel activity at negative potentials [21]. 

Spinocerebellar ataxia type 6 (SCA6) is an autosomal dominant disorder characterized by a late onset slowly progressive ataxia, dysarthria and nystagmus associated with an abnormal CAG expansion in exon 47 of CACNA1A gene [22]. The normal number of CAG repeats ranges up to 18 and individuals with SCA6 have 20 to 33 repeats in the carboxy-terminal domain, resulting in a polyglutamine expansion of the carboxyl- terminal domain [22].

### Episodic Ataxia Type 3 (EA3)

EA3 (MIM 606554) was described in 26 members of a single large Canadian family with episodic vertigo, tinnitus, ataxia, migraine and interictal myokimia [23]. Patients respond to acetazolamide and no residual ataxia was observed after the episodes. The disease locus for EA3 has been mapped to chromosome 1q42 [24].

### Episodic Ataxia Type 4 (EA4)

EA4, also called familial periodic vestibulocerebellar ataxia (MIM 606552), is an autosomal dominant disorder characterized by episodes of vertigo and ataxia beginning in the third to sixth decade of life described in two families of North Caroline, USA [25, 26]. Patients may have interictal nystagmus and mild ataxia similar to EA2 or they may be completely normal in between attacks. The episodes usually last for hours and are not relieved by acetazolamide. The most consistent symptom is the inability to suppress the vestibulo-ocular reflex. Linkage analysis ruled out the EA1 and EA2 loci as well as loci for SCA1–5 [27], but the locus has not been identified.

### Episodic Ataxia Type 5 (EA5)

EA5 (MIM601949) was identified when a series of families with episodic ataxia and epilepsy harboured mutations in the calcium channel b4 subunit CACNB4, on chromosome 2q22-23 [28]. The premature-termination mutation R482X was identiﬁed in a patient with juvenile myoclonic epilepsy. The R482X protein lacks the 38 C-terminal amino acids containing part of an interaction domain for the subunit. The missense mutation C104F was discovered both in a German family with generalized epilepsy and praxis-induced seizures and in a Canadian family with episodic ataxia. These coding mutations were not detected in unaffected controls [29].

### Episodic Ataxia Type 6 (EA6)

EA6 (MIM612656) was first identified in a 10-year-old child with an episodic and progressive ataxia, seizures, alternating hemiparesis and episodes of migraine [30]. A de-novo P290R heterozygous mutation was identified by candidate gene approach in SLC1A3 (MIM 600111). This is a solute carrier gene which encodes the glial excitatory amino acid transporter type 1 (EAAT1), which is involved in glutamate removal from the synapses [30]. A second mutation C186S was identified in 3 symptomatic members of a family with EA, without motor symptoms or seizures, suggesting a relationship between the phenotype and the extent of glutamate transporter dysfunction [31].

### Episodic Ataxia Type 7 (EA7)

EA7 (MIM611907) was described in a family of 7 members with vertigo, weakness, dysarthria and ataxia lasting from hours to days typically triggered by exercise or excitement, with onset before age 20 [32]. There is no interictal finding (which distinguishes it from EA3). Genome scan mapped the locus of EA7 between rs1366444 and rs952108 on chromosome 19q13.

## VESTIBULAR MIGRAINE (VM)

Migraine is an episodic headache disorder affecting 8% of males and 17% of females [33]. Clinically, the International Classification of Headache Disorders (ICHD-II) [34] recognizes two main common forms of migraine: migraine with aura and migraine without aura. The two forms are distinguished from each other based on the presence of aura, a period of variable and diverse neurological symptoms that precedes the headache phase. There is a dilemma among the scientific community whether migraine with aura and migraine without aura attacks represent two different disorders or if they are variations of a single disease having a common complex genetic background.

The clinical criteria required for diagnosing VM are current or previous history of migraine according to ICHD-II criteria [34] and at least 2 attacks of vertigo presenting with 1 of the following symptoms: migrainous headache, photophobia, phonophobia, or visual or other auras. Vertigo is not considered to be an aura of migraine [35]. VM is found in 1-3% of the population, being the most common cause of recurrent vertigo. Migraine is a complex polygenic disease and this may explain clinical heterogeneity (migraine, aura or vertigo). 

A recent genome-wide association study of 2731 European migraine patients has identified rs1835740 on chromosome 8q22.1 (P = 5.38 × 1 10^−9^, odds ratio = 1 1.23, 95% CI 1 1.150–1.324). The association was replicated in 3,202 cases and 40,062 controls [2]. The rs1835740 is located between MTDH (astrocyte elevated gene 1, also known as AEG-1) and PGCP (encoding plasma glutamate carboxypeptidase). In an expression study in lymphoblastoid cell lines, transcript levels of the MTDH were found to have a significant correlation with rs1835740 minor allele. MTDH downregulates SLC1A2, the gene encoding the major glutamate transporter (EAAT2) in the brain [36, 37], suggesting that the identified variant regulates glutamate in the synapsis. In another population-based genome-wide analysis including 5122 migraineurs and 18108 non-migraineurs individuals, rs2651899 (1p36.32, PRDM16) rs10166942 (2q37.1, TRPM8) and rs11172113 (12q13.3, LRP1) were among the top markers associated with migraine. The meta-analysis of three replication cohorts including 774 from Netherlands, 306 from Germany and 2748 from the International Headache Genetics Consortium confirmed the association when discovery and replication cohorts were pooled [3]. TRPM8 encodes a sensor for cold and cold-induced pain and is a target for neuropathic pain [38]. The potential role of PRDM16 in migraine is unclear. LRP1 is a member of the lipoprotein family which modulates synaptic transmission and co-localizes with NMDA receptor in neurons [39], supporting that glutamate homeostasis is relevant in the pathophysiology of migraine. Future challenges should be a GWAS in VM patients, since this is a subtype of migraine patients to define endophenotypes and a fine mapping analysis of MTDH, TRPM8 and LRP1 genes in VM.

### Familial Migranous Vertigo/Familial VM

In 1994, a set of three families with multiple members who experienced migraine and episodes of vertigo lasting minutes followed years later by progressive loss of peripheral vestibular function was reported [40]. A 4-generation family with 23 members with vestibular migraine inherited as a dominant trait has been described [41]. Members of this family have episodic vertigo and migraine with aura. A genome-wide screen for loci linked to MV segregating in this large family and subsequent fine structure mapping demonstrated that the disease gene is located between loci rs244895 and D5S2073 in chromosome 5q35. Candidate genes in this region, including KCNMB1 (Kv channel interacting protein 1 isoform 1), KCNIP1 (potassium large conductance calcium activated), ATP6V0E (ATPase, H+ transporting, lysosomal, V0 subunit E), SLC34A1 (solute carrier family 34 sodium phosphate), GABRP (gamma-amino butyric acid-GABA A receptor); DRD1 (dopamine receptor D1); and HRH2 (histamine receptor H2) were sequenced, but no mutation was identified [41]. 

## BILATERAL VESTIBULAR HYPOFUNCTION (BVH)

A small family with recurrent vertigo without migraine neither hearing loss was described in 2003 [42]. The episodes of vertigo were triggered by exercise and stress, and patients showed improvement with acetazolamide. In the later stages, when the frequency of vertigo attacks diminished, the patients developed imbalance and oscillopsia. In contrast to the large number of deafness genes, no mutations have been identified in BVH with normal hearing. Improving of phenotyping of patients with recurrent vertigo and BVH, excluding ototoxicity is essential to investigate their genome. BVH is usually progressive and it is observed after longer periods of follow up, usually in subjects over 60 years-old, making difficult the recruitment of patients within a family from more than two generations. Moreover, selection of sporadic cases with BVH also requires long follow-up periods to confirm BVH.

There has been a single report of linkage analysis in families with a dominantly inherited bilateral vestibulopathy syndrome associated with migraine and normal hearing [43]. The preliminary analysis showed genetic heterogeneity. Since the human inner ear transcriptome has been obtained, collaborative efforts in identifying and recruiting patients with familial vestibulopathy will help identify genes by high throughput sequencing methods.

## MENIERE DISEASE

Meniere’s disease (MD [MIM 156000]) is defined as an inner ear disorder characterized initially by fluctuating low-frequency sensorineural hearing loss, recurrent vertigo attacks, aural fullness, and tinnitus. Patients experience a high frequency of vertigo attacks during the first years which decrease over time and develop progressive imbalance and severe profound hearing loss [44]. The second ear could be affected in around 15-40% of cases. A disturbance of the cochlear fluid homeostasis and the endocochlear potential has been involved in the development of MD [45], and endolymphatic hydrops has been consistently identified as a histological feature in temporal bone specimens from patients with MD.

MD is a complex genetic disorder and several features of MD suggest a genetic or epigenetic component. The prevalence of the disease is more common in European Caucasians than in Asian or African populations. Familial cases represent 4-20% of all patients and several MD pedigrees have been reported, mostly in Caucasians [46, 47].

### Familial Meniere’s Disease (FMD)

A familial history of MD has been described in 10-19% of cases (Table **2**). The inheritance of MD has been reported to be autosomal dominant with incomplete penetrance estimated around 60% [48-50]. Anticipation for FMD, dependent upon the number of MD patients per generation has been described in several families, including both earlier onset and tendency to more severe symptoms in successive generations [51-54]. In addition, several Brazilian families with concurrent MD and migraine have also been described [55].

A candidate region for FMD was identified on chromosome 12p12.3 from linkage analysis in three large Swedish families [56]. This region was analyzed in 15 families with several cases segregating FMD in at least two patients. The confirmed allelic association defines an haplotype in 12p12.3 between the markers D12S373 and GT27 [57]. Two genes are located in the region: RERG/RAS-like gene (RERGL) and PIK3C2G gene; however, none of these genes show any alterations in the coding sequences in patients with FMD. In contrast, two non-coding SNPs close to exon 29 of PIK3C2G, rs12827507 and rs11044211 were strongly associated with FMD in Swedish families [57].

Another study in 19 German families with 52 members affected by FMD (27% bilateral FMD) found linkage with a region on chromosome 5 (LOD score =1.9) and 13/19 families showed consistent linkage to the region identified by D5S644, which contains 105 known genes [47]. The association observed in these families replicates an earlier linkage analysis using data from different families. Moreover, these families had a prevalence of migraine around 39% and presented an earlier onset in the third and fourth generation.

FMD has also been investigated in Finland in 8 families with 17 individuals with definite FMD [58]. The inheritance was autosomal dominant and the majority of patients were women, a finding also observed in British and German patients with FMD [47, 49]. A recent study in 16 Finnish families did not confirm anticipation, co segregation with migraine or linkage to the 12p12.3 region found in Swedish FMD, suggesting genetic heterogeneity within FMD [58].

Massive parallel sequencing of the whole exoma in selected cases of FMD is an emerging high-throughput method that will facilitate the discovery of new genes by eliminating the need for linkage analysis and candidate gene screening [59].

### Sporadic Meniere’s Disease

A candidate gene approach, either by case-control studies or by the sequencing of selected genes, has been used to search genetic markers associated with MD (Table **3**). These genes include antiquitin [60], aquaporin 2 [61], COCH (*coagulation factor C homology*) [62], potassium channels genes (KCNE1 and KCNE3) [63], MHC class II genes [64, 65], alpha-adducin [66], heat-shock protein 70 [67], PARP-1 [68], PTPN22 [69], Fc gamma receptors CD32 and CD16a [70] and nitric oxide synthases type 1 and 2 [71]. However, none of these genes could be replicated in an independent set of MD patients [72-74]. 

The HapMap3 Project has defined 10 million single nucleotide polymorphisms (SNPs) throughout the human genome providing markers for a detailed analysis in any chromosomal region, predicting that an SNP will be in linkage disequilibrium to the gene or mutation of interest. The combination of efficient techniques for high throughput genotyping with dense SNP maps offers a high level of definition for genome-wide association studies.

SNP-based scans using biallelic polymorphisms are most likely to detect common sequence variants associated with disease development than candidate gene or familial linkage approaches. The effect size of these common variants has been small (odds ratio, <2), and a common genetic variant will not account for the entire heritability of MD. This observation leads to investigations of rare variants, copy number variation, and epigenetic modifications to explain the entire heritability of a given disease [75, 76]. Rare structural variants are suspected to account for the molecular differences underlying varied clinical phenotypes and they will be associated with FMD.

Ultimately, definition of allelic variants for MD will need confirmation through experimental modeling demonstrating physiologic dysfunction. Discovery of a mechanism of disease development will advance accuracy of the diagnosis and refinement of clinical phenotypes, and provide new targets for pharmacologic intervention. Currently, an international consortium, named GENOMEN, has been organized to define the clinical phenotype and to investigate common and rare variants in 2000 European Caucasian adults with MD, to identify genetic markers for the disease and define its biochemical pathways.

## Figures and Tables

**Table 1:** Genetic and Clinical Summary of Episodic Ataxia Syndromes

EA Type	Age at onset, y	Duration of episodes	Associated symptoms	Interictal findings	Gene locus	Gene
EA1	<20	Minutes	Muscle spams	Myokimia, seizures	12p13	KCNA1
EA2	<20	Hours	Vertigo, weakness	Ataxia, nystagmus	19p13	CACNA1A
EA3	<20	Minutes	Vertigo, tinnitus, headache	No	1q42	Unknown
EA4	20-50	Hours	Vertigo, diplopia	Nystagmus, abnormal smooth pursuit	Unknown	Unknown
EA5	20-60	Hours	Vertigo	Nystagmus, ataxia	2q22-23	CACNB4
EA6	<10	Hours	Cognitive impairment	Seizures, ataxia	5p13	SLC1A3
EA7	<20	Hours	Vertigo	No	19q13	Unknown

**Table 2:** Linkage Association Studies in Familial Ménière’s Disease

Gene locus	Ethnic	Candidate gene	Anticipation	Replication	Phenotype
12p12.3	Swedish	PIK3C2G	Y	No	MD, migraine
14q11-11	UK	None	Y	No	MD, migraine
DFNA9 - 14p11.2	Belgium, Netherland	COCH	Y	Y	Sensorineural hearing loss and vestibular dysfunction
5	German	None	?	No	MD, migraine. Bilateral 27%
Unknown	Brazilian	None	?	No	MD and migraine
Unknown	Finnish	None	No	No	MD
1q32.1-32.3	Chilean- North Spain	SCLA45A3	?	No	MD

**Table 3:** History of Candidate Gene Association Studies in Ménière’s disease. None of them were Replicated in an Independent Population

Gene	Paper	Phenotype	Cases	Controls	Odds ratio	P value
HLA-DRB1*1201	Koyama S *et al.* 1993	Sporadic MD	20	…	…	0,04
COCH	Fransen E *et al.* 1999	Familial MD	23	119	…	…
HLA-DRB1*09	Meng X *et al.* 2001	Sporadic MD	60	85	0,2	0,01
Antiquitin	Lynch M *et al.* 2002	Familial MD	9	…	…	…
HLA-Cw*07	Melchiorri L *et al.* 2002	Sporadic MD	41	101	3,6	6,9.E^-3^
HLA-DRB1*0405	Koo JW *et al.* 2003	Sporadic MD	41	226	8,51	0,006
KCNE1	Doi K *et al.* 2005	Sporadic MD	63	205	NC	NC
KCNE3	Doi K *et al.* 2005	Sporadic MD	63	237	NC	NC
HLA-DRB1*1101	Lopez-Escamez JA *et al.* 2007	Sporadic MD	80	250	3,65	0,029
Alpha-adducin	Teggi R *et al.* 2008	Sporadic MD	28	48	…	0,0034
HSP 70	Kawaguchi S *et al.* 2008	Sporadic MD	49	100	…	<0,001
PARP-1	Lopez-Escamez JA *et al.* 2009	Bilateral MD	80	371	7,33	0,012
PTPN22	Lopez-Escamez JA *et al.* 2010	Bilateral MD	52	348	2,25	0,04
FcγRIIIa	Lopez-Escamez JA *et al.* 2011	Sporadic MD	156	626	1,49	0,054
NOS1/NOS2A*	Gazquez I *et al.*, 2011	Sporadic MD	381	667	0.89	0.56

* A CCTTT microsatellite was initially associated in Galicia population, OR=0.37, corrected p=0.04.
